# Activity of Fengycin and Iturin A Isolated From *Bacillus subtilis* Z-14 on *Gaeumannomyces graminis* Var. *tritici* and Soil Microbial Diversity

**DOI:** 10.3389/fmicb.2021.682437

**Published:** 2021-06-18

**Authors:** Jiawen Xiao, Xiaojun Guo, Xinlei Qiao, Xuechao Zhang, Xiaomeng Chen, Dongdong Zhang

**Affiliations:** College of Life Science, Hebei Agricultural University, Baoding, China

**Keywords:** wheat take-all, *Bacillus subtilis*, fengycin, iturin A, soil microbial diversity

## Abstract

*Bacillus subtilis* Z-14 can inhibit phytopathogenic fungi, and is used as a biocontrol agent for wheat take-all disease. The present study used the soil-borne fungus *Gaeumannomyces graminis* var. *tritici* (*Ggt*), which causes wheat take-all disease, and the soil microbial community as indicators, and investigated the antifungal effects of fengycin and iturin A purified from strain Z-14 using high performance liquid chromatography and matrix-assisted laser desorption/ionization time-of-flight mass spectrometry, respectively. The results showed that fengycin destroyed the internal structure of *Ggt* cells by digesting the cytoplasm and organelles, forming vacuoles, and inducing hyphal shrinkage and distortion. Iturin A induced cell wall disappearance, membrane degeneration, intracellular material shrinkage, and hyphal fragmentation. A biocontrol test demonstrated a 100% control effect on wheat take-all when wheat seedlings were treated with fengycin at 100 μg/ml or iturin A at 500 μg/ml. Iturin A and fengycin both reduced the relative abundance of *Aspergillus* and *Gibberella*. At the genus level, iturin A reduced the relative abundance of *Mortierella* and *Myrothecium*, while fengycin reduced that of *Fusarium.* Only fengycin treatment for 7 days had a significant effect on soil bacterial diversity.

## Introduction

The fungus *Gaeumannomyces graminis* (Sacc.) Arx & Olivier var. *tritici* J. Walker (*Ggt*), a soil pathogen, causes take-all, an important wheat (*Triticum aestivum* L.) root disease worldwide, which seriously affects the wheat grain quality and causes 60% yield reduction or even complete failure of the yield (Gutteridge et al., 2003; Keenan et al., 2015). Currently, there is no wheat variety that is resistant to *Ggt*, and traditional crop rotation cannot be achieved easily in large-scale planted crops such as wheat. Meanwhile, chemical pesticides are ineffective against take-all disease and generally cause environmental pollution and pathogen resistance (Paulitz et al., 2010). However, alternative biocontrol strategies using naturally existing antagonistic microorganisms for plant disease prevention are considered environmentally friendly and ecologically sound (Huang et al., 2012).

Recently, the use of *Bacillus* species as effective biological control agents against soil-borne diseases has received increasing research attention (Yánez-Mendizábal et al., 2012; Liu et al., 2015). The endospores formed by the *Bacillus* genus endow them with strong heat-resistance and desiccation-tolerance, resulting in their development as easily stored commercial products with a long shelf life (Posada et al., 2016). *Bacillus* strains are distributed widely in soil, plant microflora, and plants. When colonizing the plant rhizosphere, they produce compounds that act as antibiotics with broad spectrum activity against fungi, which can suppress various plant pathogens (Stein, 2005). The antifungal compounds produced by *Bacillus* isolates comprise volatiles (Gao et al., 2018), antibiotics (Liu et al., 2014), peptides, and proteins (Zhang X. et al., 2013; Kim et al., 2015). In particular, the effects of three kinds of small (1–1.5 kDa) lipopeptides (surfactins, iturins, and fengycins) on plant fungal pathogens have been investigated (Ongena and Jacques, 2008). *B. subtilis* NCD-2 strongly inhibits many kinds of phytopathogenic fungi. Matrix-assisted laser desorption/ionization time-of-flight mass spectrometry (MALDI-TOF MS) identified that the antifungal activity could be attributed to a group of fengycin homologs (Guo et al., 2014). The aflatoxin-producing fungi *Aspergillus parasiticus* and *Aspergillus flavus* were strongly inhibited by *B. pumilus* HY1 isolated from kanjang (Korean soybean sauce) *via* an antifungal compound similar to the known lipopeptide, iturin (Cho et al., 2009). Antagonism caused by the secretion of a variety of antifungal substances is one of the main biological control mechanisms of *Bacillus* isolates, among which lipopeptide antibiotics synthesized by the non-ribosome pathway play a significant role. *Bacillu*s-secreted lipopeptides, such as iturins and fengycins, strongly antagonize a broad spectrum of plant pathogenic fungi, but rarely inhibit bacteria (Cawoy et al., 2015; Chen et al., 2019). These *Bacillus*-secreted antifungal lipopeptides cause hyphal deformity, fracture, condensation of protoplasm, and inhibition of spore germination of plant pathogenic fungi (Ratón et al., 2012). However, the differences between these two lipopeptides in terms of their inhibitory mechanisms against pathogenic fungi and their effect on soil microbial diversity remain unclear.

The control effect of living microbial agents is often affected by the environment, which can fluctuate substantially in different years or different regions (Akum et al., 2020). Using lipopeptides directly to control plant diseases is not economical at present. However, as research on their synthetic regulatory mechanism and the yield optimization of lipopeptides progresses (Dang et al., 2019; Zhang et al., 2021), it will become increasingly realistic to use lipopeptides to control plant diseases to compensate for the deficiencies of using intact cells (Chakraborty et al., 2020; Kourmentza et al., 2021).

A biocontrol bacterium that was isolated from soil of the wheat rhizosphere, *B. subtilis* Z-14, exhibits a wide range of antifungal abilities against various phytopathogenic fungi, including strong growth inhibition of *Ggt* (Zhang et al., 2017a). Methanol extracts of strain Z-14 fermentation supernatant were shown to contain three lipopeptide antibiotic families (surfactins, iturins, and fengycins). The combination of isolation using high performance liquid chromatography (HPLC), antifungal activity measurement *via* a diffusion plate assay, and component detection using MALDI-TOF MS analysis demonstrated that iturins and fengycins were the main antifungal metabolites of strain Z-14 (Zhang et al., 2021). The present study aimed to determine the differences in the antifungal mechanisms against the phytopathogenic fungus of iturins and fengycins purified from strain Z-14 fermentation supernatant, and their inhibitory spectrum against fungi and bacteria, using the wheat take-all pathogen and all the microorganisms in soil as indicators.

## Materials and Methods

### Strains

The *Ggt* antagonistic bacterium *B. subtilis* Z-14 was stored on streak-inoculating nutritive-agar slants at 4°C (Zhang et al., 2017a). A virulent isolate of *Ggt*, AnH8, was kindly donated by Professor Kejian Ding of the School of Plant Protection, Anhui Agricultural University, China. AnH8 was stored at 4°C on a potato dextrose agar (PDA) slant. For both test and control samples, a 5-mm-diameter AnH8 mycelial disc was placed in the center of PDA plates. These plates were then incubated for 7 days at 25°C in the dark.

### Soil Samples and Wheat Seeds

On Feb 9th, 2018, a non-sterile soil sample was collected from a layer of soil 5–15 cm in depth originating from the Hebei Agricultural University’s experimental field on which wheat was grown continuously for 6 years. This study used Winter wheat seeds (cultivar shi4185), which were preserved by the research center of crop germplasm resources of Hebei Agricultural University, China. Shi4185 has never been treated using fungicides.

### Lipopeptide Extraction From the Fermentation Supernatant of *Bacillus subtilis* Z-14

A 37°C, nutrient agar (NA) culture of *B. subtilis* strain Z-14 was transferred into an Erlenmeyer flask with 50 ml of seed culture medium. The flask was shaken at 220 rpm and 37°C for 48 h. The culture was centrifuged at 10,000 × *g* for 15 min to collect the supernatant, which was filtered through a 0.22 μm Millipore filter (Millipore, Billerica, MA, United States) (Chen et al., 2019). Hydrochloric acid (6 mol/l) was used to adjust the pH of the filtrate to pH 2 before overnight storage at 4°C. The filtrates were then centrifuged for 20 min at 8,000 × *g* to recover the precipitate. The precipitate was washed twice using a diluted HCl solution (pH 2) and then extracted twice using methanol (Kim et al., 2015). Before HPLC and MALDI-TOF mass spectrometry analyses, the extract was filtered through a 0.22 μm hydrophobic membrane. A portion of the extract was dried using a rotary vacuum evaporator and then resuspended in an equivalent volume of sterile water to detect its antifungal activity against *Ggt*.

### Isolation and Detection of Lipopeptides Produced by *Bacillus subtilis* Z-14

The antifungal substances contained in the crude extract were isolated by HPLC using a 1260 series instrument (Agilent, Santa Clara, CA, United States) equipped with a C18 reversed-phase column (150 mm × 4.6 mm, 5 μm). After consulting the literature and exploring the separation conditions, the optimum separation conditions were determined as follows: The mobile phase comprised (A) 0.1% trifluoroacetic acid in water and (B) acetonitrile (60:40), a flow rate of 0.8 ml/min, the column temperature was 25°C, and a UV detector was employed at a detection wavelength of 230 nm. The eluent of each peak was collected in an Eppendorf tube and the corresponding retention time was recorded. The eluents were vacuum dried and dissolved in sterile water for subsequent activity detection. Eluents possessing antifungal activity against *Ggt* were analyzed using a 5800 MALDI-TOF mass spectrometer (AB SCIEX, Redwood, WA, United States) employed in positive reflectron mode with a matrix comprising α-cyano-4-hydroxycinnamic acid (Velho et al., 2011). Isolated and identified lipopeptides from the culture supernatant of strain Z-14 were assessed by HPLC to determine their purity using the parameter settings described above.

### Electron Microscopic Examination of the Inhibitory Effect of Lipopeptides Against *Gaeumannomyces graminis* var. *tritici*

The antifungal activities of the isolated lipopeptides were determined *via* a diffusion plate assay according to a previously described method, with some modifications (Pretorius et al., 2015). Four 7-mm diameter, evenly-spaced wells were created at 2.5 cm from the center of a PDA plate containing streptomycin sulfate (40 μg/ml). A sterile water control or an aliquot of the lipopeptide solutions (all 50 μl) were added to the wells and a *Ggt* fungus plug was placed at the center of the plate. The plates were incubated at 25°C until the antifungal zone of inhibition could be observed. The experiment was repeated in triplicate. The fungal colonies were excised or mycelial samples were taken from the edges of the antifungal zones and their morphologies were observed using an S-3500N scanning electron microscope (SEM) (Hitachi, Tokyo, Japan) and ultrastructure variations were observed under a H-7650 transmission electron microscope (TEM) (Hitachi), as described previously (Chen et al., 2014).

### Fluorescence Microscopic Examination of the Inhibitory Effect of Lipopeptides Against *Gaeumannomyces graminis var. tritici*

The cell viability assay was performed using fluorescein diacetate (FDA) stain and propidium iodide (PI) stain (Gu et al., 2017). The culture plates were incubated at 25°C for 7 days until the antifungal inhibition zone appeared, then sterilized coverslips were tilted into the antifungal zone and cultured for 3–5 days before fluorescence staining was performed for observation. Then, 10 μl of FDA or PI molecular probes were added to the coverslips, and the cell suspensions were incubated for 15 min at 25°C in dark. The samples were detected using an Olympus BX43 microscope, and findings were analyzed using cellSens standard software (Olympus, Tokyo, Japan).

### Assay of the 50% Inhibitory Concentrations of Purified Iturin A and Fengycin Against *Gaeumannomyces graminis* var. *tritici*

To determine the 50% inhibitory concentrations (IC50) of purified iturin A and fengycin against *Ggt* hypha, three doses (60, 120, and 180 μg) of iturin A or fengycin were added separately to three aliquots each containing 4 ml PDA medium at 45°C, mixed rapidly and poured into three separate small petri dishes. After the agar had cooled down, a single 5-mm diameter mycelial disc of the fungus *Ggt* was placed in the center of each plate. Sterile water only served as the control. All the plates were cultured at 25°C until the mycelia colony of the control had grown to almost fill the plate. The area of the mycelia colony was measured, and the inhibition of fungal growth in the other plates was determined by calculating the percent reduction in the area of the mycelia colony (Liu et al., 2010).

### Treatment of Wheat Take-all by Fengycin and Iturin A in Petri Dishes

Fengycin and iturin A, respectively, were made up as solutions at different concentrations (10, 50, 100, and 500 μg/ml) and stored for subsequent use. After cultivation of the wheat take-all pathogen for 7 days, a mycelial cake with a diameter of 7 mm was prepared for later use. A previously described method was used to determine the inhibitory ability of the crude extract against *Ggt*-induced wheat take-all (Zhang et al., 2017a). Soaking in 70% ethanol for 90 s was used to sterilize wheat seeds, which were then rinsed thrice using sterilized water, before being germinated under aseptic conditions at 30°C. When the root length of each seed was greater than 10 mm, germinated seeds (*n* = 10) were placed evenly on filter papers in 20-cm diameter dishes and subjected to different treatments: Aliquots (10 ml) of sterile water only as a blank control (C0); sterile water + *Ggt* as the pathogen control (C1); *Ggt* + fengycin group: 100 μl of different concentrations (A1, 10 μg/ml; A2, 50 μg/ml; A3, 100 μg/ml; A4, 500 μg/ml) of fengycin solution was added to each wheat seed; *Ggt* + iturin A group: 100 μl of different concentrations (B1, 10 μg/ml; B2, 50 μg/ml; B3, 100 μg/ml; B4, 500 μg/ml) of iturin A solution was added to each wheat seed. For pathogen inoculation, a fresh 7-mm diameter mycelial disk of *Ggt* was placed directly on the wheat seminal roots. The dishes were incubated for 10 days at room temperature, with the filter papers being kept moist. The wheat plants were then harvested carefully and evaluated for the occurrence and severity of take-all disease. Previously described formulae were used to determine disease reduction (DR) and the disease index (DI) (Zhang et al., 2017b). In addition, the shoot heights, root lengths, and fresh shoot and root weights were determined. Each treatment was performed using three replicates, and the experiment was repeated three times.

### Soil Sample Processing

The soil was sieved using a 40 mesh sieve to remove impurities and large soil blocks, and then divided into five treatment groups. Three replicates were included for each treatment. One treatment labeled as CK0 (CK01, CK02, and CK03) was stored directly at −80°C without any management. Two treatments labeled as CK1 (CK11, CK12, and CK13) and CK2 (CK21, CK22, and CK23) were incubated at 25°C for 7 and 14 days, respectively. Two treatments labeled as Fen1 (Fen11, Fen12, and Fen13) and Fen2 (Fen21, Fen22, and Fen23) comprised soil mixed with purified fengycin at a final concentration as 50 μg/g of soil and incubated at 25°C for 7 and 14 days, respectively. The last two treatments, labeled as Itu1 (Itu11, Itu12, and Itu13) and Itu2 (Itu21, Itu22, and Itu23), comprised soil mixed with purified C14 iturin A at a final concentration as 50 μg/g of soil and incubated at 25°C for 7 and 14 days, respectively.

### Illumina HiSeq Sequencing of the Soil Sample DNA

DNA was extracted from approximately 500 mg of each treated soil sample using an E.Z.N.A.^®^ soil DNA Isolation Kit (Omega Bio-tek, Norcross, GA, United States) according to the manufacturer’s protocol. A Qubit 3.0 Fluorometer (Invitrogen, Grand Island, NY, United States) was used to quantify the DNA concentrations of all samples. 16S rRNA gene sequencing was used for bacterial profiling, in which the bacterial 16S gene V4 region was amplified using PCR primers 515F (5′-GTGCCAGCMGCCGCGGTAA-3′) and 806R (5′-GGACTACHVGGGTW TCTAAT-3′) (Caporaso et al., 2011). Modified primers ITS1F (5′-CTTGGTCATTTAGAGG AAGTAA-3′) and ITS2 (5′-GCTGCGTTCTTCATCGATGC-3′) were used to amplify the first fungal internal transcribed spacer (ITS1) region (Elizabeth et al., 2018). PCR reaction systems for amplification of the bacterial V4 region and fungal ITS1 region consisted of 1 μl of genomic DNA, 4 μl of 5 × FastPfu PCR buffer, 0.4 mmol/l of each dNTP, 1 μmol/l of each primer, 0.3% bovine serum albumin (New England Biolabs, Hitchin, United Kingdom), and 0.4 μl of FastPfu polymerase to make up a 20-μl reaction volume for the PCR reaction. Using a PCR System DNA thermal cycler (Bio-Rad, Hercules, CA, United States), amplification was conducted under the following conditions: 2 min at 94°C; followed by 30 cycles of 30 s at 94°C, 30 s at 50°C, and 30 s at 72°C; and a final incubation for 10 min at 72°C. The amplicons were visualized using gel electrophoresis in an ethidium bromide stained 1.0% agarose gel to check that they were the correct size and lacked contamination, followed by gel purification using a GeneJET Gel Extraction Kit (Thermo Fisher Scientific, Waltham, MA, United States). The 16S and ITS1 amplicons were sequenced by Biomarker Biotechnology Co., Ltd. (Beijing, China) using the Illumina HiSeq 2500 sequencing system (Illumina, San Diego, CA, United States) (Qiao et al., 2018).

### Processing of High-Throughput Sequencing Data

FLASH v.1.2.7 was used to merge the terminal sequence data obtained from Hiseq sequencing into raw tags, based on the overlap between paired-end reads. The raw tags were filtered with Trimmomatic v0.33 and chimeric sequences was identified and removed using UCHIME v4.2 to obtain the finial effective Tags (Hu et al., 2018). The Quantitative Insights Into Microbial Ecology (QIIME 1.8.0) toolkit was then used to analyze the sequencing data (Caporaso et al., 2010b). Clustering of the optimized sequences into operational taxonomic units (OTUs) at the 97% sequence similarity level was performed using UCLUST (Edgar, 2010). The Ribosomal Database Project (RDP) classifier with the SILVA databases was used for taxonomic assignments of bacterial OTU representative sequences (Zhang et al., 2018), while standalone Mega BLAST searches of the UNITE database were used to assign the fungal OTU sequences (Abarenkov et al., 2010).

### Statistical Analysis

The taxonomic analysis of the samples was carried out at each taxonomic level based on the results of OTU analysis, and a species distribution histogram was obtained at different classification levels (Caporaso et al., 2010a). The alpha diversity indices, including Chao1, ACE, Shannon, and Simpson, were calculated using Mothur version v.1.30 and the summary single command (Grice et al., 2009). Principal coordinates analysis (PCoA) was performed using the R software package (version 2.15.3) based on the relative abundance of fungal and bacterial genera (Sakaki et al., 1994). Data from replicates are expressed as the mean ± standard deviation (SD). The Statistical Product and Service Solutions (SPSS) v.17.0 software package (IBM Corp., Armonk, NY, United States) was used to perform the calculations and compare the treatment means for each experiment. Significant differences between the means were assessed using Duncan’s tests and one-way analysis of variance (ANOVA). Statistical significance was accepted at *p* ≤ 0.05.

## Results and Discussion

### Ingredient and Purity Detection of Iturin A and Fengycin Secreted by *Bacillus subtilis* Strain Z-14

**FIGURE 1:**
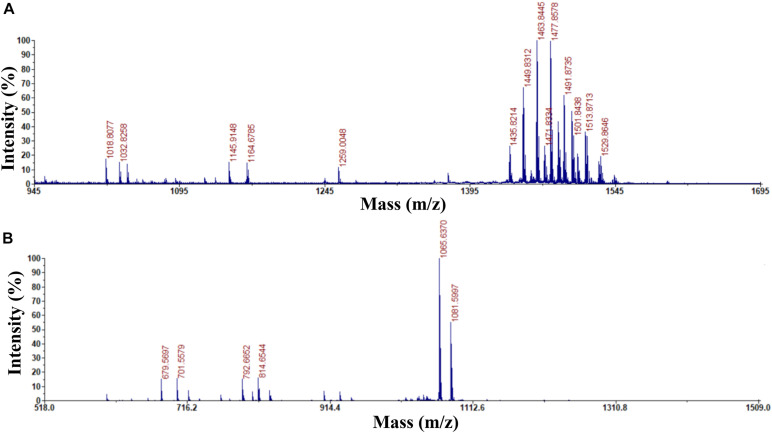
Determination of the purity and ingredients of fengycin and C14 iturin A by matrix assisted laser desorption ionization-time of flight mass spectrometry. **(A)** The purity and ingredient analysis for the eluent with a retention time of 2.736 min isolated by HPLC and identified as fengycin; **(B)** the purity and ingredient analysis for the eluent with a retention time of 11.218 min isolated by HPLC and identified as C14 iturin A.

Previous studies used HPLC and MALDI-TOF-MS to extract, purify, and detect fengycin and iturin A from *B. subtilis* BS155 and *B. amyloliquefaciens* S76-3 (Gong et al., 2015; Zhang and Sun, 2018). HPLC purification and MALDI-TOF-MS structural identification analysis of *B. subtilis* Z-14 fermentation supernatant crude extract resulted in the isolation and detection of fengycin and iturin A. For the eluent with a retention time of 2.736 min, peaks appeared at m/z 1,435.8214, 1,449.8312, 1,463.8445, 1,477.8578, 1,491.8735, 1,501.8438, 1,513.8713, and 1,529.8646 Da, which, combined with several varieties of positive ions such as H^+^, Na^+^, and K^+^, corresponded to a series of homologous molecules with similar m/z values to fengycins (Figure 1A). The m/z of the molecular ion peaks in the eluents with a retention time of 11.218 min were 1,065.6370 and 1,081.5997, which corresponded to molecules of C14 iturin A, combined with positive ions of Na^+^ and K^+^, respectively (Figure 1B). Meanwhile, C15 iturin A and C16 iturin A were both completely separated by HPLC with the optimized mobile phase, which was more advanced than that described in earlier studies. For example, Kinsella et al. (2009) used two different mobile phases to separate fengycins and iturins; however, separation of iturins of different carbon chain lengths was not achieved. C14 iturin A was the main constituent of the iturin A homologs in the supernatant of strain Z-14 and was therefore used as the experimental material for subsequent experiments. The purified samples of C14 iturin A and fengycins presented single peaks in the HPLC chromatograms, which demonstrated that they were both chromatographically pure compounds. Fengycin homologs of different molecular weights could not be separated by HPLC, which agreed with the results of a previous report (Pretorius et al., 2015).

### Influence of Fengycin and Iturin A on *Gaeumannomyces graminis* var. *tritici* Growth Under Electron Microscopy

**FIGURE 2:**
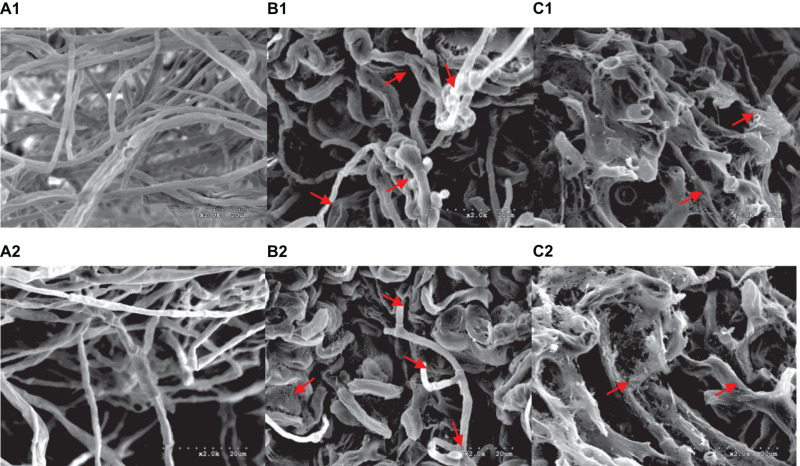
Morphological changes of *G. graminis* var. *tritici* hyphae treated with fengycin and iturin A observed using scanning electron microscopy (SEM). **(A_1_,A_2_)** The untreated *Ggt* hyphae; **(B_1_,B_2_)** the *Ggt* hyphae treated with fengycin, showing dehydration, entanglement, irregular expansion, and ring formation; **(C_1_,C_2_)** the *Ggt* hyphae treated with iturin A, showing cell membrane digestion and hyphal rupture.

**FIGURE 3:**
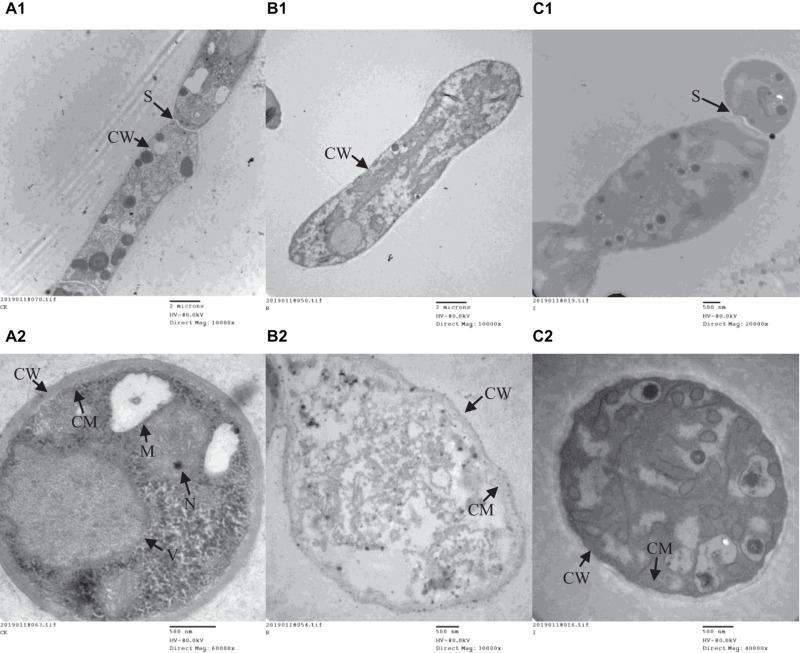
The ultrastructure of *G. graminis* var. *tritici* hyphae cells treated with fengycin and iturin A observed using transmission electron microscopy (TEM). **(A_1_,A_2_)** The untreated *Ggt* hyphae; **(B_1_,B_2_)** the *Ggt* hyphae treated with fengycin, showing cytoplasmic digestion, disintegration of organelles, and formation of a large number of blank areas; **(C_1_,C_2_)** the *Ggt* hyphae treated with iturin A, showing cell wall digestion, cell membrane degradation, and cytoplasmic pyknosis. **(A_1_,B_1_,C_1_)** longitudinal sections of *Ggt* hyphae; **(A_2_,B_2_,C_2_)** transverse sections of *Ggt* hyphae. CW, cell wall; CM, cell membrane; M, mitochondria; N, nucleus; S, septum; V, vacuole.

A range of concentrations was tested to determine the IC50 of purified iturin A and fengycin against *Ggt*. The results showed that the inhibitory activity increased with the concentration of iturin A and fengycin, and the IC50 of iturin A and fengycin were calculated as approximately 34.7 and 26.5 μg/ml, respectively. These results are consistent with the findings of Gu et al. (2017), which suggested that the IC50 of purified bacillomycin D against *Fusarium graminearum* was approximately 30 μg/ml. Like other organisms, fungal cells must continuously absorb nutrients from the outside to provide the necessary energy for regulated growth and reproduction *via* the synthesis and catabolism of intracellular organelles. Therefore, whether a pathogen can maintain its normal growth and development depends largely on the integrity of its cell structure. Therefore, SEM and TEM were used to observe the cell structure of *Ggt* treated with fengycin and iturin A isolated from Z-14 strain fermentation supernatant. A diffusion plate assay demonstrated that the inhibition zone of iturin A and fengycin against *Ggt* hyphae became obvious after 5 days of culture and the mycelial samples from the edges of the inhibition zones were taken for SEM and TEM detection. SEM observation showed that fengycin and iturin A severely affected hyphal growth and the ultrastructure of *Ggt in vitro*. The untreated normal *Ggt* hyphae appeared slender and smooth, and the cell wall was intact (Figure 2A). By contrast, the hyphae of the cells treated with fengycin became severely wrinkled and twisted into masses (Figure 2B); some hyphae became abnormally swollen or shriveled; and some hyphae twined into loops or formed blunt circular structures at the tips. *Ggt* hyphae treated with iturin A were seriously twisted and ruptured to form a rough cell surface (Figure 2C). We observed that the cell wall and membrane of most hyphae were disrupted, such that the hyphae were broken into irregular fragments. Comparison of the hyphae treated with fengycin and iturin A showed that the fengycin-treated hyphae appeared twisted and swollen and finally died; however, hyphal fracture was rare and the external morphology remained intact. In contrast, the iturin A-treated hyphae were broken into fragments because of serious damage to cell structures. Significant variations in the hyphal outer layer structure implied dramatic changes of the interior subcellular organelles, which required further examination. The effects of fengycin and iturin A on the internal structure of hyphal cells were recorded under TEM examination, which confirmed the radical changes in the internal structure of the cells (Figure 3). The untreated hyphae presented regular sections; consistent cell wall thickness; continuous cell membrane; clear nucleoli in the nuclear membrane; intact mitochondria, vacuoles, and other organelles; uniform cytoplasmic distribution; and a clear and visible diaphragm. Fengycin-treated hyphae had an irregular section surface, which indicated hyphal deformation. The texture of the cell wall was loose and thin, with blurred hyphal contours. Fengycin caused deformation or degeneration of cell membranes, digestion of the cytoplasm, disintegration of organelle, and the formation of a large number of vacuoles. When treated with iturin A, the cell wall of *Ggt* hyphae almost disappeared and the cell membrane degenerated. The intracellular materials appeared to have shrunk, forming a large number of vacuoles, with a damaged diaphragm, and broken or dysmorphic hyphae. A comparison between fengycin and iturin A-treated *Ggt* hyphae under TEM showed that the internal structure of fengycin-treated cells had been completely dissolved, while the cell wall and cell membrane remained intact despite partial degeneration. The iturin-treated cell internal structure only underwent solid condensation without complete digestion; however, the cell wall and cell membrane were completely degraded. We speculated that fengycin mainly destroyed the internal structure of *Ggt* hyphae, thus inhibiting mycelial growth, while iturin A almost destroyed the cell wall and cell membrane to antagonize *Ggt* growth. TEM and SEM observations in a previous study suggested that secreted antifungal factors in the culture supernatant of *B. amyloliquefaciens* DH-4 disrupted the *Penicillium digitatum* cellular ultrastructure. Macrolactin, bacillaene, iturins, fengycin, and surfactin were isolated and identified using ultra performance liquid chromatography electrospray ionization mass spectrometry (UPLC-EIS-MS) analysis; however, the influence of antifungal substances on the ultrastructure of *P. digitatum* cells was detected using the culture filtrate without separation (Chen et al., 2018). SEM and TEM detected and confirmed the ultrastructural changes in the hyphae of *Sclerotinia sclerotiorum*, including curling plasmolysis, shrinkage, pore formation, and hyphal breakdown caused by fengycin produced by *B. amyloliquefaciens* FZB42 (Farzand et al., 2019). Although our results were consistent with those of the above studies, our observations of the contrast between the effects of fengycin and iturin A on the ultrastructure of hyphal cells have not been reported previously.

### Cell Staining of *Gaeumannomyces graminis* var. *tritici* Treated With Fengycin and Iturin A

**FIGURE 4:**
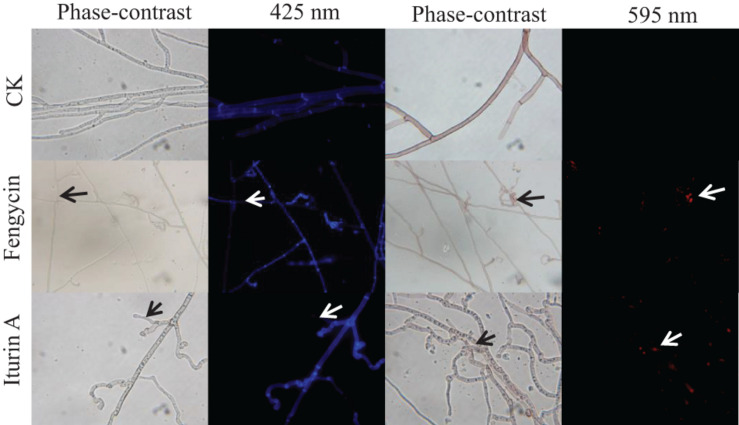
Detection of *G. graminis* var. *tritici* hyphae viability based on fluorescein diacetate (FDA) and propidium iodide (PI) staining after treatment with Fengycin and Iturin A for 7 days. Live fungal cells with intact membranes show blue fluorescence, and fungal cells with damaged membranes show red fluorescence; sterile water served as the control (CK). The damaged sites of the fungal cells membrane are labeled using arrows. The magnification was 1,000×.

To detect whether cell death occurred, FDA and PI fluorescence staining were used to detect the antifungal activity of fengycin and iturin A against *G. graminis* var. *tritici* in combination with phase-contrast and fluorescence microscopy. FDA is an enzyme activity probe that is recognized by non-specific esterases, which then releases fluorescence when it enters living cells, thus serving as an indicator of intracellular enzymatic activity (Zhao et al., 2014). PI is an analog of ethidium bromide, which can be used as a nuclear staining reagent for the detection of apoptosis (Gu et al., 2017). As shown in Figure 4, the untreated normal *Ggt* hyphae had few dead cells on the basis of no red fluorescence with PI staining. The intact cell membrane morphology could be seen in the uniform blue fluorescence profile of the untreated *Ggt* hyphae, which had even surfaces and equal widths upon FDA staining. In contrast, treatment with 50 μg/ml fengycin or iturin A caused substantially deformed and damaged morphology of *Ggt* hyphae. The blue fluorescence distribution of the *Ggt* hyphae was uneven, and the fluorescence of some hyphae was faint. The nuclear and cell membrane of the lateral tiny hyphae and the curled part at the end of the hyphae showed red fluorescence upon fengycin treatment, indicating damage to the hyphael membranes, leading to cell apoptosis seriously. Hyphae treated with iturin A displayed substantial abnormalities, such as severe distortion and shriveling. According to the FDA staining results, iturin A resulted in interrupted fluorescence along the hyphae, severe coiling of the ends of the lateral branches of the hyphae and the mycelia branch at both ends of the same node. Meanwhile, the nuclei of the microlateral hyphae showed red fluorescence. According to the results of double fluorescence staining, iturin A led to abnormal branching patterns of hyphae, and the cell membrane of lateral branches was dissolved and obviously broken, and cell apoptosis was detected. Similar results were observed for other lipopeptides. Optical and fluorescence microscopy analyses revealed severe morphological changes in conidia and substantial distortions in *F. graminearum* hyphae treated with plipastatin A (Gong et al., 2015). Analyses using scanning and transmission electron microscopy revealed that bacillomycin D caused morphological changes in the plasma membranes and cell walls of *F. graminearum* hyphae and conidia (Gu et al., 2017). Combined with the results of SEM and TEM detection, staining images suggested that both fengycin and iturin A were able to severely damage the cell wall and the cell membranes, thereby inhibiting cell growth.

### Fengycin and Iturin A Control of Wheat Take-All in Petri Dishes

**FIGURE 5:**
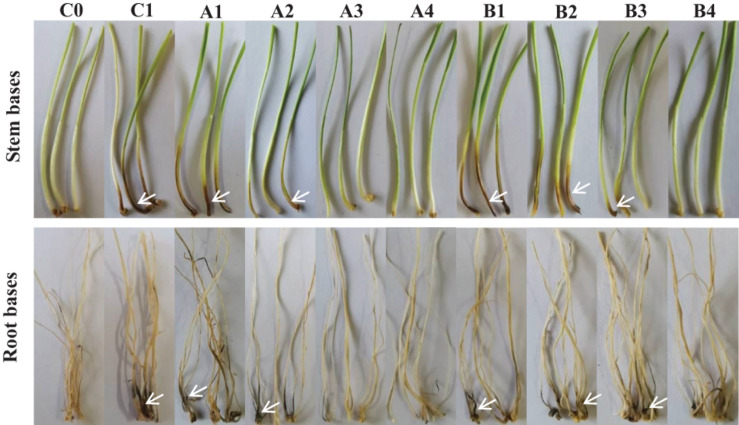
Biocontrol effect of different concentrations of fengycin and iturin A on take-all disease of wheat seedlings. C0, blank control; C1, pathogen control; A1∼A4, the effect of different concentrations of fengycin (10, 50, 100, and 500 μg/ml, respectively) on take-all disease of wheat seedlings; B1∼B4, the effect of different concentrations of iturin A (10, 50, 100, and 500 μg/ml, respectively) on take-all disease of wheat seedlings. The white arrows point to the tissues (stem bases and root bases, respectively) of wheat seedlings infected by pathogen *G. graminis* var. *tritici*.

The biocontrol efficacy of purified fengycin and iturin A isolated from the fermentation supernatant of strain Z-14 against *Gg*t-induced wheat take-all was tested in Petri dishes (Figure 5). Wheat seedlings infested by the take-all pathogen showed significant lesions on their roots and stem bases. These lesions on wheat tissues were effectively reduced by varying degrees after treatment of *Ggt*-infested seedlings with different concentrations of fengycin. The lesion symptoms on the roots and stem bases of seedlings was ameliorated in a fengycin concentration-dependent manner. According to the data shown in Table 1, the control efficacy against wheat take-all disease reached 54.44% in *Ggt*-infested seedlings treated with 10 μg/ml fengycin, while the control efficacy reached 100% at a fengycin concentration of 100 μg/ml. Wheat seedlings treated with fengycin at 100 μg/ml or above showed increased root length and shoot height, but showed no significant difference in root and shoot fresh weight compared with those of C0. Iturin A treatment of *Ggt*-infested wheat at different concentrations gradually reduced the incidence of wheat take-all. The control efficacy of iturin A against wheat take-all reached 63.89% at 50 μg/ml, and 100% control efficacy was achieved at 500 μg/ml iturin A. Iturin A had no significant growth promoting effect on root length, root weight, shoot height, and the weight of the wheat seedling. The utilization of purified lipopeptides for the *in vivo* prevention of plant diseases infested by pathogenic fungi has not been reported, and it does not represent a practical application considering the economic value. The purposes of this *in vivo* test were to verify the effectiveness and to identify the differences in function of the purified iturin A and fengycin against wheat take-all disease caused by *Ggt*. The result showed that fengycin played a more significant role in controlling *Ggt*-induced wheat take-all than did iturin A, although they both exhibited effective biocontrol activities. There are few reports of biocontrol experiments using purified lipopeptides to control plant fungal disease; however, some studies were performed using fermentation supernatants of antagonistic bacteria to prevent the invasion of phytopathogens. The culture filtrate of *B. amyloliquefaciens* strain DH-4, with the main antifungal substances identified as lipopeptides, exhibited activity against *P. digitatum*, which causes postharvest rot of citrus fruit *in vitro* and *in vivo* (Chen et al., 2018). For four *Bacillus* species, their culture filtrates and extracts all showed strong biocontrol activity against the gray mold on strawberry, grape, and tomatoes, respectively, in which lipopeptides were the main antifungal substances (Chen et al., 2019). Many biocontrol bacteria produce iturin A and fengycin simultaneously, and it is difficult to identify the main antifungal substance using the fermentation supernatant as the detection object (Zhang et al., 2017b; Chen et al., 2019). Isolation of iturin A and fengycin from the culture and the subsequent detection of the antagonistic activities *in vitro*, coupled with small scale testing *in vivo*, might represent a feasible solution.

**TABLE 1:** Biocontrol effect of different concentrations of fengycin and iturin A on wheat take-all disease.

Tr	RL (cm)	RFW (g × 10^–3^)	SH (cm)	SFW (g × 10^–3^)	DI	DR (%)
CK0	10.33 ± 1.26^d^	50.00 ± 9.00^a^	9.33 ± 1.89^c^	167.04 ± 6.24^a^	–	–
CK1	6.67 ± 2.57^bc^	47.67 ± 3.21^a^	7.02 ± 1.00^c^	140.01 ± 3.21^*bcd*^	90.02 ± 2.65^a^	–
A1	12.33 ± 2.08^ab^	28.33 ± 5.51^d^	9.33 ± 1.53^c^	137.06 ± 7.02^de^	41.01 ± 3.55^c^	54.44
A2	12.67 ± 2.31^ab^	29.66 ± 4.04^cd^	9.83 ± 3.18^c^	141.67 ± 41.86^cd^	12.50 ± 1.43^f^	86.11
A3	13.67 ± 0.58^ab^	30.33 ± 1.15^cd^	12.50 ± 2.65^a^	151.33 ± 7.09^abc^	0.00 ± 0.01^g^	100
A4	14.50 ± 1.50^a^	43.67 ± 10.01^ab^	11.33 ± 2.52^ab^	184.33 ± 1123^ab^	0.00 ± 0.01^g^	100
B1	12.51 ± 2.29^abc^	41.06 ± 3.46^ab^	8.51 ± 2.18^c^	111.01 ± 1.00^e^	75.02 ± 2.54^b^	16.67
B2	11.02 ± 2.65^abc^	38.00 ± 3.61^bc^	7.83 ± 0.76^c^	106.67 ± 5.77^e^	32.51 ± 2.13^d^	63.89
B3	10.01 ± 1.00^cd^	47.00 ± 2.65^ab^	7.83 ± 0.76^c^	131.33 ± 4.62^bcd^	17.52 ± 0.15^e^	80.56
B4	12.67 ± 1.15^ab^	44.67 ± 5.03^ab^	9.67 ± 1.15^bc^	137.33 ± 4.04^bcd^	0.00 ± 0.01^g^	100

*C0, blank control; C1, pathogen control; A, fengycin; B, iturin A; 1∼4, different concentrations of fengycin and iturin A (10, 50, 100, and 500 μg/ml, respectively). Tr, treatment; RL, root length; RFW, root fresh weight; SH, shoot height; SFW, shoot fresh weight; DI, disease index; DR, disease reduction. Data are means ± SD. Different letters in the same column indicate a significant difference at the P < 0.05 level using the least significant difference (LSD) test.*

### Effects of Fengycin and Iturin A on the Abundance and Diversity of Soil Microbial Communities

The soil microbial community, which plays an important role in soil substance circulation, ecological balance, and nutrient transformation of plants, is an important component of the soil ecosystem (Mendes et al., 2011). Understanding the effects of iturin A and fengycin on soil microbial diversity would be helpful to comprehend the mechanism of action of *Bacillus* strains in soil and provide references for other ecosystems such as plant leaves and fruit. Illumina HiSeq high-throughput sequencing of the ITS1 regions of fungal genomes from 21 soil samples produced a total of 1,126,331 reads. The double-end reads were spliced and filtered to generate 1,055,053 clean tags. Each of the samples generated at least 36,118 clean tags, with an average of 50,241 clean tags per sample. Clustering identified 698 OTUs (Table 2). Sequencing of the V4 regions of bacterial genomes from soil samples produced 1,605,073 paired reads. The paired reads were processed to obtain 1,342,641 clean tags, with at least 51,268 Clean tags for each sample and an average of 63,935 clean tags per sample. Clustering produced 1,864 OTUs (Table 3).

**TABLE 2:** OTU abundance and diversity index of fungal communities in all soil samples.

	OTU	ACE	Chao1	Simpson	Shannon
CK0	411 ± 16.97	450.30 ± 19.55^a^	477.14 ± 9.69^a^	0.22 ± 0.04^a^	2.93 ± 0.19^c^
CK1	396 ± 41.14	403.91 ± 51.22^ab^	404.17 ± 51.50^b^	0.037 ± 0.02^b^	4.32 ± 0.38^ab^
CK2	371.33 ± 34.38	397.33 ± 49.12^ab^	402.10 ± 55.73^b^	0.05 ± 0.01^b^	3.84 ± 0.17^ab^
Itu1	399.33 ± 30.02	416.65 ± 35.93^ab^	423.85 ± 45.00^ab^	0.14 ± 0.17^ab^	3.59 ± 1.01^bc^
Itu2	375.5 ± 12.02	379.83 ± 8.05^b^	381.03 ± 5.70^b^	0.04 ± 0.02^b^	4.03 ± 0.34^ab^
Fen1	381 ± 8.48	382.95 ± 8.50^b^	383.95 ± 8.26^b^	0.02 ± 0.00^b^	4.55 ± 0.00^a^
Fen2	417.333 ± 3 2.95	430.67 ± 39.80^ab^	432.13 ± 37.88^ab^	0.05 ± 0.03^b^	4.10 ± 0.41^ab^

*CK0, soil without treatment; CK1 and CK2, soil incubated at 25°C for 7 and 14 days, respectively; Itu1 and Itu2, soil treated with iturin A at 25°C for 7 and 14 days, respectively; Fen1 and Fen2, soil treated with fengycin at 25°C for 7 and 14 days, respectively. Data are means ± SD. Different letters in the same column indicate significant difference at P < 0.05 level by the least significant difference (LSD) test.*

**TABLE 3:** OTU abundance and diversity index of bacterial communities in all soil samples.

	OTU	ACE	Chao1	Simpson	Shannon
CK0	1,753.00	1,783.22 ± 2.71^a^	1,797.42 ± 2.74^a^	0.00 ± 0.00^b^	6.46 ± 0.02^a^
CK1	1,602.67	1,630.62 ± 68.26^c^	1,639.13 ± 70.49^c^	0.01 ± 0.01^a^	6.14 ± 0.08^e^
CK2	1,674.67	1,706.11 ± 75.32^bc^	1,714.44 ± 74.96^bc^	0.01 ± 0.00^b^	6.26 ± 0.05^cd^
Itu1	1,719.67	1,756.49 ± 10.67^ab^	1,772.31 ± 20.15^ab^	0.01 ± 0.00^b^	6.33 ± 0.02^bc^
Itu2	1,697.50	1,722.55 ± 14.09^ab^	1,734.72 ± 8.78^ab^	0.00 ± 0.00^b^	6.39 ± 0.07^ab^
Fen1	1,722.00	1,764.96 ± 2.02^ab^	1,775.43 ± 2.27^ab^	0.01 ± 0.00^ab^	6.17 ± 0.10^de^
Fen2	1,665.67	1,699.01 ± 51.03^bc^	1,710.26 ± 44.49^bc^	0.01 ± 0.00^ab^	6.21 ± 0.11^de^

*CK0, soil without treatment; CK1 and CK2, soil incubated at 25°C for 7 and 14 days, respectively; Itu1 and Itu2, soil treated with iturin A at 25°C for 7 and 14 days, respectively; Fen1 and Fen2, soil treated with fengycin at 25°C for 7 and 14 days, respectively. Data are means ± SD. Different letters in the same column indicate significant difference at P < 0.05 level by the least significant difference (LSD) test.*

The richness and diversity of the fungal and bacterial communities in the soil samples were studied using Alpha diversity analysis, which included the community richness indices Chao1 and ACE, and the community evenness indices Shannon and Simpson (Wei et al., 2018). The Chao1 and ACE indices of fungal diversity in CK1 and CK2 decreased significantly, but no significant difference was observed between CK1 and CK2 (Table 2). The richness of the fungal community in Itu2 was reduced significantly compared with that of CK2. Iturin A decreased the species richness of fungal diversity significantly after a 14-day trial. The species richness of fungi decreased significantly in Fen1 compared with that of CK1, but there was little difference between Fen2 and CK2. The Shannon index of fungal diversity in CK1 and CK2 increased compared with that of CK0, but showed no significant difference between CK1 and CK2. The species diversity of fungi in Itu1 increased significantly compared with that in CK1, while there was no significant difference between CK2 and Itu2. The Shannon index of fungal diversity in Fen1 increased significantly compared with that of CK1 and Itu1, but no significant difference appeared between Fen2, Itu2, and CK2, which demonstrated that fengycin had a stronger impact on fungal diversity than iturin A over a short time course (7 days). The Simpson index showed the same trends.

The Chao1 and ACE indices of bacterial diversity decreased significantly in CK1 and CK2 compared with that in CK0, but did not change significantly between CK1 and CK2, which indicated that the species abundance of bacteria decreased when the soil sample was placed at 25°C for 7 days or more (Table 3). The richness of the bacterial community did not change significantly between Itu1 and CK1, or Itu2 and CK2. In general, there was no significant change in the bacterial community in response to iturin A treatment compared with that of the control. The Chao1 and ACE indexes of the bacterial community in Fen1 were significantly higher than those of CK1, but were not significantly different between Fen2 and CK2. Meanwhile, there was no significant difference in the Shannon and Simpson indices for the bacterial community in response to fengycin or iturin A treatment compared with those of the control. Iturin A was assumed to have a finite or even no antibacterial activity, and Kourmentza et al. (2021) stated that both gram-positive and gram-negative bacteria were found not to be susceptible to lipopeptide mycosubtilin (iturin family). By contrast, fengycin demonstrated weak antibacterial activity and inhibited the growth of *Escherichia coli*, which was used as an indicator to optimize the antimicrobial activity of fengycin in milk in a response surface method (Huang et al., 2008). In the present study, we found that iturin A did not affect the soil bacterial diversity whereas fengycin had a weak impact on the bacterial diversity, which was consistent with the findings of previous studies.

### Effects of Fengycin and Iturin A on Soil Microbial Community Structure

**FIGURE 6:**
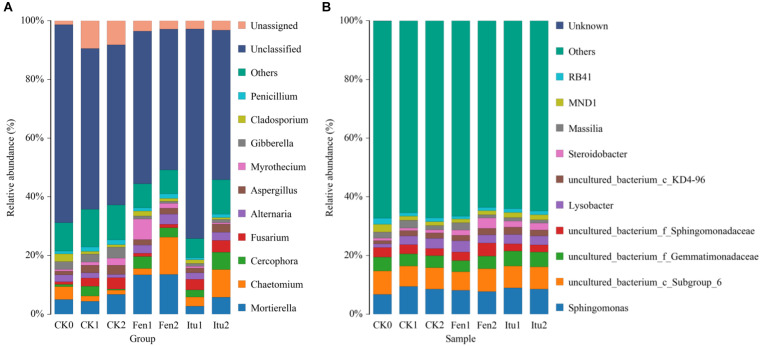
The relative abundance of the fungal **(A)** and bacterial **(B)** community structure of soil samples at the genus level.

The main groups of fungi in soil samples were *Mortierella*, *Chaetomium*, *Cercophora*, *Fusarium*, *Alternaria*, *Aspergillus*, *Myrothecium*, *Gibberella*, *Cladosporium*, and *Penicillium* at the genus level (Figure 6A). Iturin A reduced the abundance of *Mortierella*, while fengycin increased it in Fen1 and then reached a balance. Compared with that in CK2, both iturin A and fengycin significantly increased the relative abundance of *Chaetomium* after 14 days of treatment. *Chaetomium* is an important resource fungus and is prominent in controlling plant diseases (Li et al., 2011; Wang et al., 2012). The increase in the relative abundance of *Chaetomium* was considered to be beneficial to control plant diseases. *Chaetomium globosum* significantly suppressed the mycelial growth of numerous phytopathogenic fungi, especially *Setosphaeria turcica*, which causes northern corn leaf blight, an important and potentially destructive corn foliar disease (Zhang G. et al., 2013). The *Ginkgo biloba* endophytic fungus, *Chaetomium globosum* CDW7, showed marked inhibitory effects against plant pathogenic fungi, and its fermentation broth successfully inhibited the development of disease in *Sclerotinia sclerotiorum*-infected rape, providing 57.8% protective efficiency *in vivo* (Zhao et al., 2017). Iturin A and fengycin increased the relative abundance of *Chaetomium*, which proved that iturin A and fengycin could increase the number of beneficial fungi in soil to enhance the control effect. Both iturin A and fengycin increased the relative abundance of *Cercophora*, with the largest effect being contributed by iturin A. Iturin A had little effect on the relative abundance of *Fusarium*, which infects a variety of cash crops and causes scab in rice, wheat, and corn (Arie, 2019; Tinia et al., 2020); while fengycin decreased *Fusarium* levels in the soil. Both iturin A and fengycin reduced the relative abundance of *Aspergillus* and *Gibberella*. *Aspergillus* not only causes huge losses to agricultural production, but also has a harmful effect on the products *via* metabolic substance such as aflatoxins, which are the most toxic mycotoxins and are extremely harmful to human health (Li et al., 2019; Parish et al., 2019). *Gibberella* is a parasitic and pathogenic fungus of certain plants, and causes a variety of plant diseases, such as rice and corn ear rot (Parker et al., 2017). The presence of *Aspergillus* and *Gibberella* could seriously affect the quality and the yield of agricultural products. Iturin A and fengycin reduced the relative abundance of *Aspergillus* and *Gibberella*, indicating that they could both be used to control plant diseases caused by the genera *Aspergillus* and *Gibberella*. The abundance of *Myrothecium* only showed a modest change under iturin A treatment, varying by only 0.65% during the whole treatment process. However, fengycin increased the abundance of *Myrothecium* when the soil samples were treated for 7 days. The genus *Myrothecium* infects various plants, such as soybean, lentils, eggplant, pepper, and tomato, causing basal stem rot, rhizome rot, and ring rot diseases (Silva et al., 2014; Wang et al., 2014). Therefore, we speculated that iturin A and fengycin had different focuses in terms of their control of plant fungal diseases.

The main groups of bacteria in the soil samples were *Sphingomonas*, *Lysobacter*, *Steroidobacter*, and *Massilia*. Iturin A had no significant effect on soil bacterial diversity at the genus level. The relative abundance of *Sphingomonas* increased from 6.76% (CK0) to 9.49% (CK1) and then decreased to 8.50% (CK2), while it increased to 8.06% in Fen1, but decreased to 7.74% in Fen2. Fengycin reduced the abundance of *Sphingomonas*, but increased that of *Steroidobacter*. The relative abundance of *Steroidobacter* increased from 0.83% (CK0) to 0.97% (CK1) and then to 1.06% (CK2), but to 1.79% in Fen1 and 3.56% in Fen2 (Figure 6B). The reduction of the abundance of *Sphingomonas* verified the results of a previous report, which showed that fengycin has weak antibacterial activity (Huang et al., 2008); however, the growth promoting effect of fengycin on bacteria has not been reported. Surprisingly, the abundance of *Steroidobacter* increased when the soil was treated with fengycin, which was speculated to be caused by the inhibition of some bacteria by fengycin, which indirectly promoted the proliferation of other genera.

### Evaluation of the Beta Diversity of Microbial Communities in Soil Samples

**FIGURE 7:**
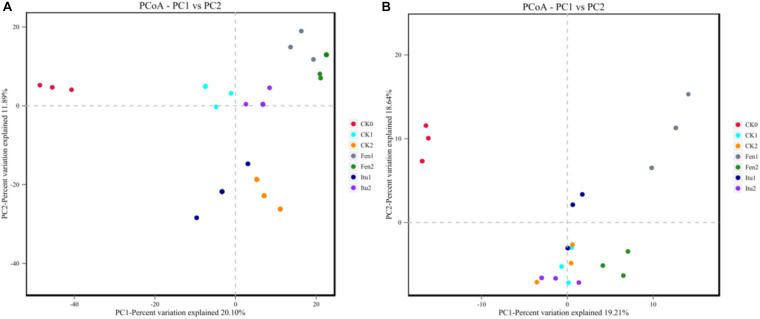
Principal component analysis of fungal **(A)** and bacterial **(B)** communities from soil samples.

Principal coordinate analysis was used to classify multiple samples and further illustrate the differences in species diversity among the samples. Based on the four distance matrices obtained from Beta diversity analysis, the PCoA analysis results were drawn using R language tools (Zhang et al., 2018). There were significant differences in the fungal flora structure among the control treatments, and also significant differences between iturin A or fengycin treatments and CK0 (Figure 7A). The difference was significant between Itu1 and Itu2, but not between Fen1 and Fen2. Both iturin A and fengycin changed the soil fungal diversity; however, the effect of iturin A on soil fungal diversity was greatly affected by the treatment time, whereas the effect of fengycin basically reached a balance after 7 days of treatment. The difference in the soil bacterial flora structure between CK0 and CK1 was significant but was not significant between CK1 and CK2. The bacterial flora structure of Itu1 and Itu2 was significantly different from that of CK0; however, there was no significant difference between Itu1 and CK1, or Itu2 and CK2. This indicated that iturin A had little effect on the soil bacterial diversity. The bacterial flora structure of Fen1 and Fen2 was significantly different from that of CK0, and the difference between Fen1 and CK1 was also significant, which demonstrated that fengycin had a significant effect on the soil bacterial diversity when the soil was treated for 7 days (Figure 7B).

## Conclusion

Fengycins and C14 iturin A were isolated and identified from the supernatant of *Bacillus subtilis* strain Z-14. Fengycins and iturin A used different methods to inhibit the growth of *Ggt*. Fengycin mainly destroyed the internal structure of *Ggt* hyphae, while iturin A almost destroyed the cell wall and cell membrane to inhibit fungal growth. Meanwhile, Fengycin demonstrated a stronger biocontrol effect against wheat take-all disease than iturin A in petri dishes. Both iturin A and fengycin reduced the amount of harmful fungi in soil. Nevertheless, fengycin had significant effect on soil bacterial diversity, but iturin A did not. This study revealed the antagonistic mechanism of iturin A and fengycin using different indicators (a single pathogen isolate *Ggt* and the complex soil microecosystem), in different culture conditions (*in vitro*, *in vivo*, and in the soil environment), which provides theoretical support and a material basis for the prevention and treatment of fungal diseases by the application of lipopeptide antibiotics.

## Data Availability Statement

The raw data supporting the conclusions of this article will be made available by the authors, without undue reservation.

## Author Contributions

DZ and XG conceived and designed the study. DZ, XZ, and XC wrote the manuscript. JX, XZ, XQ, and XC performed the experiments. XG, XZ, and XC performed the statistical analyses. All authors contributed to the article and approved the submitted version.

## Conflict of Interest

The authors declare that the research was conducted in the absence of any commercial or financial relationships that could be construed as a potential conflict of interest.
